# Supplementary material for: Electrocardiogram-based diagnosis of liver diseases: an externally validated and explainable machine learning approach
Source: eClinicalMedicine. 2025 May 20;84:103242. doi: 10.1016/j.eclinm.2025.103242 (PMC12149549; doi:10.1016/j.eclinm.2025.103242)
Supplement: Supplementary Tables S3–S5 [file mmc1.docx]

# Supplementary Material

**Additional results from the internal dataset**

Table [3](#_bookmark0) presents the predictive performance of our model across various ICD-10 codes from the internal dataset (MIMIC-IV-ECG), including conditions that were not available in the external validation dataset. Notably, the highest-performing conditions tend to be chronic hep- atic diseases such as alcohol-induced chronic pancreati- tis (K860, AUROC = 0.9609) and alcoholic hepatitis with ascites (K7011, AUROC = 0.9033). However, the table also includes several acute or subacute conditions, such as alcohol-induced acute pancreatitis (K852, AUROC = 0.9255) and acute and subacute hepatic failure (K720, AU- ROC = 0.7009), which allow for some assessment of the model’s applicability beyond chronic diseases. While the model generally maintains good performance across a va- riety of conditions, the results highlight the need for future studies to explore additional non-alcoholic and acute hep- atic conditions.

# Model performance comparison for ECG fea- tures and demographics

Table [4](#_bookmark2) presents the model performance (AUROC) for predicting different liver disease conditions using ECG features and demographic data. Notably, ECG-based mod- els outperform demographic-based models in predicting hepatic failure (K72, K729, K7290), with higher AUROC scores in both internal and external test sets. This suggests that ECG signals capture relevant physiological changes associated with hepatic failure. Conversely, demographic- based models show stronger performance for alcoholic liver disease (K70) and alcoholic cirrhosis (K703, K7030), indicating that patient characteristics such as age, sex, and medical history play a more significant role in these condi- tions compared to ECG features. For comparison, we also indicate the results for the models operating on the com- bined feature set as presented in the main text. The com- bined models show statistically significant improved per- formance levels, assessed per non-overlapping confidence intervals, compared to single-modality models.

# Model performance by demographic sub- groups

Table [5](#_bookmark3) presents the AUROC values for both internal and external datasets, stratified by gender and age quan- tiles. The results indicate consistent performance across different demographic groups, although certain subgroups exhibit variability, particularly in older age groups and fe- male patients with specific conditions. The stratified AU- ROC values highlight potential demographic influences

on model performance. In particular, performance varies across age quantiles, with a decrease in predictive accuracy for older patients in some conditions (e.g., K70, K703). This suggests that age-related physiological changes or variations in disease presentation may impact ECG-based predictions. Additionally, gender differences, such as higher AUROC for females in certain conditions (e.g., K7030), may reflect underlying biological or dataset dis- tribution factors.

# Hyperparameters

To ensure optimal model performance and prevent over- fitting, we employed early stopping with a patience of 10 iterations, following standard practices for gradient- boosting models. Given that XGBoost inherently retains the best iteration based on validation performance, the to- tal number of training iterations does not directly influence the final model.

We used the default hyperparameter settings of XG- Boost without explicit tuning of the number of trees, max- imum depth, or learning rate. However, we acknowledge that alternative hyperparameter configurations could im- pact model performance and may be explored in future work. The training hyperparameters for each model were as follows:

- **alpha**: 0
- **cache opt**: 1
- **colsample bylevel**: 1
- **colsample bynode**: 1
- **colsample bytree**: 1
- **eta (learning rate)**: 0.300000012
- **gamma**: 0
- **grow policy**: depthwise
- **interaction constraints**: None
- **lambda (L2 regularization)**: 1
- **max bin**: 256
- **max cat threshold**: 64
- **max cat to onehot**: 4
- **max delta step**: 0
- **max depth**: 6
- **max leaves**: 0
- **min child weight**: 1
- **min split loss**: 0
- **monotone constraints**: None
- **refresh leaf**: 1
- **reg alpha (L1 regularization)**: 0
- **reg lambda (L2 regularization)**: 1
- **sampling method**: uniform
- **sketch ratio**: 2
- **sparse threshold**: 0.2
- **subsample**: 1
- **num parallel tree**: 1
- **num trees**: Between 25 and 37 per code/model

| **ICD-10 Code** | **AUROC (95% CI)** | **Description** |
| --- | --- | --- |
| K860 | 0.9609 (0.9612, 0.9619) | Alcohol-induced chronic pancreatitis |
| K852 | 0.9255 (0.9229, 0.9272) | Alcohol-induced acute pancreatitis |
| K7011 | 0.9033 (0.9033, 0.9046) | Alcoholic hepatitis with ascites |
| K7110 | 0.8784 (0.8791, 0.8807) | Toxic liver disease with hepatic necrosis, without coma |
| K7010 | 0.8687 (0.8676, 0.8696) | Alcoholic hepatitis without ascites |
| K761 | 0.8444 (0.8489, 0.8510) | Chronic passive congestion of liver |
| K7031 | 0.8408 (0.8414, 0.8427) | Alcoholic cirrhosis of liver with ascites |
| K7681 | 0.8380 (0.8420, 0.8435) | Hepatopulmonary syndrome |
| K9423 | 0.8153 (0.8231, 0.8263) | Gastrostomy malfunction |
| K652 | 0.7972 (0.7973, 0.8008) | Spontaneous bacterial peritonitis |
| K767 | 0.7966 (0.7939, 0.7961) | Hepatorenal syndrome |
| K704 | 0.7874 (0.7782, 0.7839) | Alcoholic hepatic failure |
| K623 | 0.7840 (0.7741, 0.7836) | Rectal prolapse |
| K8689 | 0.7699 (0.7771, 0.7819) | Other specified diseases of pancreas |
| K8051 | 0.7671 (0.7655, 0.7684) | Calculus of bile duct without cholangitis or cholecystitis with obstruction |
| K2920 | 0.7659 (0.7659, 0.7698) | Alcoholic gastritis without bleeding |
| K7040 | 0.7600 (0.7522, 0.7557) | Alcoholic hepatic failure without coma |
| K711 | 0.7462 (0.7470, 0.7544) | Toxic liver disease with hepatic necrosis |
| K5732 | 0.7462 (0.7469, 0.7494) | Diverticulitis of large intestine without perforation or abscess without bleeding |
| K807 | 0.7455 (0.7368, 0.7399) | Calculus of gallbladder and bile duct without cholecystitis |
| K292 | 0.7450 (0.7476, 0.7510) | Alcoholic gastritis |
| K5669 | 0.7442 (0.7437, 0.7481) | Other intestinal obstruction |
| K3589 | 0.7421 (0.7416, 0.7462) | Other acute appendicitis |
| K758 | 0.7381 (0.7346, 0.7406) | Other specified inflammatory liver diseases |
| K264 | 0.7314 (0.7319, 0.7337) | Chronic or unspecified duodenal ulcer with hemorrhage |
| K745 | 0.7313 (0.7307, 0.7350) | Biliary cirrhosis, unspecified |
| K862 | 0.7287 (0.7211, 0.7277) | Cyst of pancreas |
| K7291 | 0.7259 (0.7188, 0.7213) | Hepatic failure, unspecified with coma |
| K7581 | 0.7212 (0.7245, 0.7291) | Nonalcoholic steatohepatitis (NASH) |
| K2210 | 0.7129 (0.7007, 0.7161) | Ulcer of esophagus without bleeding |
| K912 | 0.7099 (0.7113, 0.7154) | Postsurgical malabsorption, not elsewhere classified |
| K762 | 0.7095 (0.7094, 0.7105) | Central hemorrhagic necrosis of liver |
| K7200 | 0.7067 (0.7052, 0.7085) | Acute and subacute hepatic failure without coma |
| K804 | 0.7050 (0.7005, 0.7033) | Calculus of bile duct with cholecystitis |
| K029 | 0.7021 (0.7005, 0.7030) | Dental caries, unspecified |
| K720 | 0.7009 (0.6996, 0.7034) | Acute and subacute hepatic failure |

Table 3. Predictive performance across different ICD-10 codes from the internal dataset (MIMIC-IV-ECG), showing AUROC values for conditions that were not available in the external dataset (ECG-ViEW II).

**ICD-10 Code Diagnosis Internal Test (AUC) External Test (AUC)**

| **ECG Features Only** | | | |
| --- | --- | --- | --- |
| K70 | Alcoholic liver disease | 0.7029 | 0.6423 |
| K703 | Alcoholic cirrhosis | 0.7088 | 0.7753 |
| K7030 | Alcoholic cirrhosis without ascites | 0.6753 | 0.7790 |
| K729 | Hepatic failure, unspecified | 0.7180 | 0.7500 |
| K7290 | Hepatic failure without coma | 0.7390 | 0.8149 |
| K72 | Hepatic failure | 0.7002 | 0.7489 |
| **Demographics Only** | | | |
| K70 | Alcoholic liver disease | 0.7352 | 0.7197 |
| K703 | Alcoholic cirrhosis | 0.7157 | 0.7156 |
| K7030 | Alcoholic cirrhosis without ascites | 0.7286 | 0.7119 |
| K729 | Hepatic failure, unspecified | 0.6645 | 0.6271 |
| K7290 | Hepatic failure without coma | 0.6497 | 0.6019 |
| K72 | Hepatic failure | 0.6548 | 0.6006 |
| **Combined (see main text)** | | | |
| K70 | Alcoholic liver disease | 0.8025 | 0.7644 |
| K703 | Alcoholic cirrhosis | 0.7887 | 0.8590 |
| K7030 | Alcoholic cirrhosis without ascites | 0.7819 | 0.8777 |
| K729 | Hepatic failure, unspecified | 0.7404 | 0.7498 |
| K7290 | Hepatic failure without coma | 0.7647 | 0.7821 |
| K72 | Hepatic failure | 0.7833 | 0.8003 |

Table 4. Comparison of model performance (AUROC) for ECG-based and demographic-based predictions across internal and external test sets for different ICD-10 codes.

| **Condition (ICD-10)** | **Female** | **Male** | **Q1 (18-53/18-40)** | **Q2 (53-66/40-52)** | **Q3 (66-78/52-65)** | **Q4 (78-101/65-109)** |
| --- | --- | --- | --- | --- | --- | --- |
| K70: Alcoholic liver disease | 0.8732/0.7528 | 0.7389/0.7060 | 0.8149/0.7320 | 0.7426/0.7610 | 0.7202/0.7395 | 0.3603/0.7480 |
| K703: Alcoholic cirrhosis | 0.8538/0.8329 | 0.7234/0.8187 | 0.8059/0.9136 | 0.7206/0.8918 | 0.7153/0.8148 | 0.4422/0.7803 |
| K7030: Alcoholic cirrhosis w/o ascites | 0.8481/0.9182 | 0.7105/0.8290 | 0.7996/0.9278 | 0.7571/0.9142 | 0.7046/0.8620 | 0.3546/0.8130 |
| K72: Hepatic failure, NEC | 0.7017/0.7055 | 0.7523/0.7636 | 0.7798/0.6687 | 0.6443/0.7721 | 0.7044/0.7640 | 0.4660/0.6993 |
| K729: Hepatic failure, unspecified | 0.7394/0.7276 | 0.7721/0.8045 | 0.8408/0.7213 | 0.6798/0.7995 | 0.6169/0.7819 | 0.4266/0.7070 |
| K7290: Hepatic failure, unspecified w/o coma | 0.7647/0.7740 | 0.7841/0.8059 | 0.8568/0.6656 | 0.6328/0.8770 | 0.6968/0.7980 | 0.6569/0.7542 |

Table 5. AUROC values for internal and external datasets, stratified by gender and age quantiles.
